# 

**Published:** 1848-04

**Authors:** 


### CONTENTS.

### LIBRARY.

PAGE.

An Essay on the Structure and Formation of the Teeth in Man and Various Animals. By Robert Blake, M. D., Revised and Corrected with Notes, by C. O. Cone, D. D. S. . . . . . 41

### JOURNAL.

ARTICLE I.—A Course of Lectures on Dental Physiology and Surgery, delivered at the Middlesex Hospital School. By John Tomes, Esq., Surgeon Dentist to the Hospital, . . . . 209

ARTICLE II.—Observations upon the Importance and Value of a Knowledge of the Collateral Branches of Medicine and Surgery, in connection with Dentistry. By James Robinson, D. D. S., London, . . . . . . . . . . . . . 247

ARTICLE III.—Irregularity of the Teeth. By J. Taft, Surgeon Dentist, Xenia, Ohio, . . . . . . . . . . 253

ARTICLE IV.—Abrasion of the Teeth. By J. Taft, Surgeon Dentist, Xenia, Ohio, . . . . . . . . . . . 254

ARTICLE V.—A case of Molar and Maxillary Absorption. By Wm. A. Pease, . . . . . . . . . . . 255

ARTICLE VI.—Gutta Percha—Its Uses, &c. By G. F. J. Colburn, Dentist, Newark, N. J. . . . . . . . . . 258

ARTICLE VII.—Valedictory Address. By Eleazar Parmly, M. D. . 260

ARTICLE VIII.—Letter from W. P. Blake, New York. . . . 278

ARTICLE IX.—On Cleft Palate, and on Staphyloraphy. By William Fergusson, Esq., F. R. S. E., Professor of Surgery in King's College, London. . . . . . . . . . . 279

ARTICLE X.—Anatomical and Physiological Relations of the Mouth. By Professor W. R. Handy, M. D., Baltimore. . . . . 294

### BIBLIOGRAPHICAL NOTICES.

The Principles and Practice of Midwifery. By David H. Tucker, M. D., Professor of the Principles and Practice of Medicine, &c. 306

Elements of General Pathology. By Alfred Stille, M. D., Lecturer on Pathology and the Practice of Medicine, in the Philadelphia Medical Association, &c. . . . . . . . . . 306

A Popular Treatise on the Teeth. By Mayo G. Smith, Dental Surgeon, 307

### MISCELLANEOUS NOTICES.

Circular to the Fellows of the American Society of Dental Surgeons, and the Profession generally, . . . . . . . . . 308

Commencement of the Baltimore College of Dental Surgery, . . . 309

The Discovery of the Application of Ether for the Prevention of Pain, 310

Disease of the Lining Membrane of the Ear, . . . . . . . 311

G. W. Parmly, Dentist, . . . . . . . . . . . 311

The Removal of a Foreign Body from the Duct of Wharton, . . . 312

